# Exploring EFL learners’ acceptance and cognitive absorption at VR-Based language learning: A survey and experimental study

**DOI:** 10.1016/j.heliyon.2024.e24863

**Published:** 2024-01-20

**Authors:** Liwei Hsu

**Affiliations:** Professor, Graduate Institute of Hospitality Management National Kaohsiung University of Hospitality and Tourism, No. 1, Song-he Rd., Hsiao-kang District, Kaohsiung City, Taiwan

**Keywords:** Cognitive absorption, Hedonic motivation system adoption model, Language learning, Neurophysiological experiment, Virtual reality

## Abstract

This study aimed to explore the applicability of VR-based language learning in an EFL context. An online survey was conducted to understand the structural relationship between EFL learners' cognitive absorption, behavioral intention to use VR for English learning, and perceptions regarding the sense of immersion created by VR. The hedonic motivation system adoption model (HMSAM) was adopted, and 230 valid responses were retrieved for statistical analyses. The results showed that most constructs of HMSAM, namely, perceived ease of use, perceived usefulness, curiosity, joy, control, behavioral intention to use, and immersion, were significantly associated with other constructs. VR's immersion had a positive significant influence on the behavioral intention of EFL learners to engage in VR-based language learning. It was revealed that curiosity was not a significant predictor for immersion. Moreover, a within-subject neurophysiological experiment was conducted with 33 EFL learners who experienced both VR-based and non-VR-based settings to examine the influence of VR technologies on their cognitive absorption and learning outcomes. Results demonstrated that VR did increase the participants' cognitive absorption; furthermore, participants had better retention about the learned contents in VR-based setting. The findings have practical and theoretical implications based on the findings of the survey and experiment.

## 1 Introduction

With the expeditious progress in information communication technology (ICT), innovation and development help improve the performance of education by improving students' learning outcomes (Sok & Cass, 2015). In recent years, substantial attention has been paid to the applicability of virtual reality (VR) in educational practices [1]. More than half of academic works on the educational application of VR technologies have focused on high-immersion VR [2]. One of the most promising benefits that such technologies can contribute to learning is an authentic environment (Chen & Yuen, 2023; [3,4]. The term virtual reality was coined by Ref. [5] as “an immersive computer enabled technology that replicates an environment and allows a simulation of the user to be present and interact in that environment” (p. 222), which has attracted increasing attention from researchers and practitioners in the field of second language (L2) education [6]. Scholars have argued that VR can enhance L2 learners’ situated learning experiences [7], as it provides learners a unique opportunity to immerse themselves in a virtual environment that simulates real-life situations, making language learning more engaging and interactive [[8], [9], [10]]. Hence, VR has been shown to be an effective tool for L2 learning [2,11,12], including vocabulary learning [[13], [14], [15], [16]], oral proficiency [17], writing [9], and listening comprehension [10].

As previous studies have shown the affordances of VR in enhancing L2 teaching and learning, the optimized applicability in L2 learning remains inconclusive [10,18]. Moreover, it has been reported that VR would only cultivate language learners' lower cognitive skills, while their higher cognitive skills are always neglected by VR-based language teaching [6,19]. Whether VR is suitable to facilitate language learners' development of their overall cognitive skills remains underexplored. As such, this study aimed to investigate L2 learners' acceptance of using VR to achieve better learning outcomes and applicability of VR-based language learning in EFL settings based on learners’ cognitive absorption (CA).

CA is a psychological state of deep involvement with an activity or technology characterized by heightened enjoyment, curiosity, temporal dissociation, focused immersion, and control [20]. It is based on the concept of cognitive engagement [21] and the theory of flow [22] and has been extensively researched in various academic fields, such as psychology, education, and marketing. CA has been used as a construct to measure user engagement and immersion in various technologies [20,23], such as e-learning systems (Saadé & Bahli, 2005), online games (Hou & Li, 2014), social media platforms (Zhang et al., 2016), and VR applications (Chen et al., 2017). CA influences user attitudes and behaviors toward technology adoption and use [24]. Moreover, CA is an indicator of learners' motivation and interest in using VR as a language learning tool as CA reflects learners' cognitive processes and emotional responses when interacting with a VR environment. Therefore, this study examined the effect of CA on learners’ acceptance and experiences of VR-based language learning and their L2 learning outcomes.

The hedonic motivation system adoption model (HMSAM) is a theoretical framework that has been commonly used to explain users’ adoption of e-learning systems that satisfy their intrinsic motivation and needs (Palos-Sanchez et al., 2021). This model suggests that users experience CA, which influences how easy and fun users perceive a system to be, which affects their intention to use it. However, previous research on CA has been criticized for lacking proper instrumentation to measure and collect real-time data on individual cognition as self-report questionnaires and behavioral experiments were the primary data collection methods [25]. Therefore, this study aimed to provide a wider spectrum of VR-based language learning using a survey and neurophysiological experiment to bridge this research gap. Three research questions have been formulated:

RQ1: What are the variables that affect EFL learners’ acceptance of VR-based language learning?

RQ2: What is the CA of EFL learners when using VR-based language learning?

RQ3: How is the CA of EFL learners related to their learning outcomes in VR-based language learning?

## 2 Literature review

### 2.1 VR in L2 learning

VR has been shown to improve the learnability of target content by equipping learners with emotional support [26]; Wang et al., 2021) and lower cognitive loads [27]. Along the same stream [2], found that VR had cognitive and affective benefits for language learners [28]. asserted that VR had been considered by 30 Japanese EFL learners as the most fun and effective tool for language learning. Chen and Yuen (2023) demonstrated that VR technology motivated students to engage in learning and improved their language skills. Furthermore, the affordance of VR to lower L2 learners' anxiety has been postulated by scholars such as [29,30]. Tai and Chen (2022) revealed that virtual presence in mobile VR improved L2 learner involvement, prevented cognitive overload, reduced anxiety, and aided comprehension [26]. showed that VR boosted EFL learners’ motivation and confidence in using English for oral communication. A study by Lin et al. (2021) employed a quasi-experiment method on a group of 38 EFL learners who used VR to learn tourism English for a duration of 7 weeks (approximately 1 and a half months) and indicated that their learning outcomes did saliently improve. Despite the importance of VR technologies in L2 education, how can such technologies be optimally applied in EFL contexts has not yet been fully explored, which calls for further empirical work [9,31].

### 2.2 Cognitive absorption and VR-based language learning

CA plays a significant role in learners' academic performance in e-learning contexts [32]. CA is an intrinsic motivator that can affect an individual's trust in e-learning applications and intention to continue using them [33,34]. [35] found that CA influenced individual attitudes toward, and intention to use, and actual use of cloud-based e-learning systems. In the nutshell, by being deeply involved and engaged with e-learning materials, learners are more likely to trust and continue to use the system, leading to better learning outcomes [36]. Furthermore, learners' mental models of learning were created due to situated learning engendered by VR, and in situ learning in VR environments resulted in better learning outcomes [37] because once their CA has been raised and better learning outcomes can be achieved [20].

### 2.3 The hedonic motivation system adoption model

**Fig. 1:**
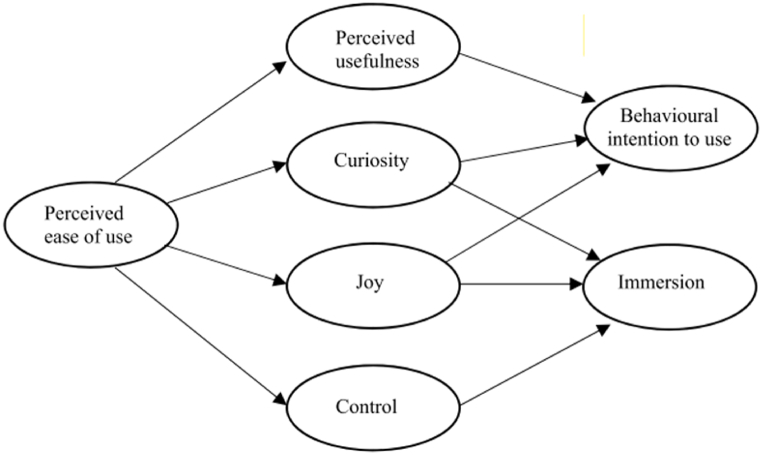
HMSAM [43].

Extending [38] hedonic system adoption model (HSAM), which was based on Davis' (1989) technology acceptance model (TAM), HMSAM sheds light on learners’ attitudes toward e-learning systems [39,40], gamified learning environment [41,42] as well as their flow experiences (i.e., concentration, intrinsic motivation, and enjoyment, please see Li et al., 2019 for details) and behavioral intention of continuous use [43]. The HMSAM helps researchers and practitioners design and evaluate target systems that enhance user engagement and satisfaction. According to Ref. [43]; seven main constructs are included in the HMSAM: perceived ease of use, perceived usefulness, curiosity, joy, control, behavioral intention to use, and immersion. Curiosity, joy, and control are “the sub-constructs of CA [that] should be conceptualized as discrete constructs” [43]; p. 631). Furthermore, it has been postulated that perceived ease of use affects perceived usefulness, curiosity (Sweetser & Weyth, 2005), joy (Merikivi et al., 2017), and control [43], and perceived usefulness, curiosity, and joy influence behavioral intention to use. Curiosity, joy, and control are the determinants of immersion. The proposed HMSAM is depicted in Fig. 1.

Many scholars have acknowledged the adequacy of HMSAM for academic research and adopted it to unpack user experiences with using innovative technology in educational practices, which covered multifaceted personal reasons such as the control of using, the ability to use, the happiness of such use and the level of immersion when using the technology [40,42]; Sampurna et al., 2021). Similarly, this study used the HMSAM to explore EFL learners’ perceptions of the hedonic and immersion functions of VR technology in English L2 learning.

## 3 Methodology

### 3.1 Materials and methods

Two studies were designed to address research questions. Study 1 addressed RQ1; whereas Study 2 addressed RQ2 and RQ3. The underlying rationale of this research method was that a survey was conducted to understand EFL learners who had prior experience using VR, but not of EFL learning yet. The aim of Study 1 was to gain a general idea about EFL learners' acceptance of using VR for EFL learning. Afterwards, some of the participants of Study 1 were invited to partake in Study 2, which was an experimental design to understand the effects of VR-based language learning for EFL learners, and doing so through the lens of EFL learners’ physiological and behavioral data.

### 3.2 Study 1

#### 3.2.1 Research procedure and participants

Study 1 adopted the HMSAM to survey EFL learners’ perceptions of the applicability of VR to EFL learning. The number of participants was calculated using G*Power (effect size *f*^*2*^ = 0.15, α error probability = .05, statistical power = .8), which suggested that a total sample of 77 would be sufficient. Study 1 recruited 230 participants (*n* = 230) through popular social media in Taiwan, such as Facebook and Line Group. Information about the recruitment was posted by the research assistants and the link to the online survey was provided along with the message. Those who were keen to join the survey could simply do so with one click. They could drop out of this survey any time they wanted by closing the webpage. Online individuals with prior experience using VR for learning or games but not specifically for EFL learning were included. Before they completed the survey, they were asked to view a video clip (https://www.youtube.com/watch?v=L6xWtv6apdM) showcasing the functionality and affordance of VR-based language learning. Based on their previous experience of using VR, they were able to understand how VR-based language learning would be implemented This online survey was conducted from December 2022 to February 2023 and the demographic information about the valid participants is reported in Table 1.

**Table 1:** Demographic information of the participants in Study 1.

Demographic background	Number of participants (*n* = 230)
Gender Males Females	102 128
Academic major Hospitality and tourism management Aviation industry management Management of information technology Age 18∼19 20∼21 21∼22 Above 23 English Proficiency (TOEIC Score) Below 545 550∼665 670∼780 Above 785	174 40 36 56 135 30 9 33 153 42 2

According to the information conveyed in Table 1, among these 230 participants, 128 (56 %) were females whereas 102 (44 %) were males. In terms of their academic majors, most of them (76 %) were from hospitality and tourism management departments followed by aviation industry management majors (18 %); the rest were management of information system majors (16 %). As for their ages, 135 (59 %) were 20–21 years old, while the number of those aged 18–19 years was 56 (24 %). Thirty of them were aged 21–22 (30 %), followed by those who were above 23 years old (9, being around 0.04 %). The TOEIC score was the standardized test used to assess their level of proficiency in English. The majority scored 550–665 (153, which was about 67 %), followed by those who achieved a 670–780 TOEIC score (42, which was about 18 %). Participants who recorded TOEIC scores below 545 or above 785 were 33 (14 %) and 2 (1 %) respectively.

As for the statistical analysis of Study 1, partial least squares structural equation modeling (PLS-SEM) was used to analyze data and identify the structural relationships between the HMSAM constructs. PLS-SEM was used due to its exploratory nature and suitability for composite structural equation modelling (SEM) (Sarstede & Hwang, 2020). Analyses were conducted using SmartPLS 4.0 to examine the following research hypotheses, which were based on the model developed and proposed by Ref. [43].

RH1 Perceived ease of use positively and significantly affects perceived usefulness in VR-based language learning.

RH2 Perceived ease of use positively and significantly affects curiosity in VR-based language learning.

RH3 Perceived ease of use positively and significantly affects control in VR-based language learning.

RH4 Perceived ease of use positively and significantly affects joy in VR-based language learning.

RH5 Perceived usefulness positively and significantly affects behavioral intention to use VR-based language learning.

RH6 Curiosity positively and significantly affects behavioral intention to use VR-based language learning.

RH7 Joy positively and significantly affects behavioral intention to use VR-based language learning.

RH8 Curiosity positively and significantly affects focused immersion in use VR-based language learning.

RH9 Joy positively and significantly affects focused immersion in VR-based language learning.

RH10 Control positively and significantly affects focused immersion in use VR-based language learning.

RH11 Focused immersion in VR-based language learning positively and significantly affects the behavioral intention to use it.

**Fig. 2:**
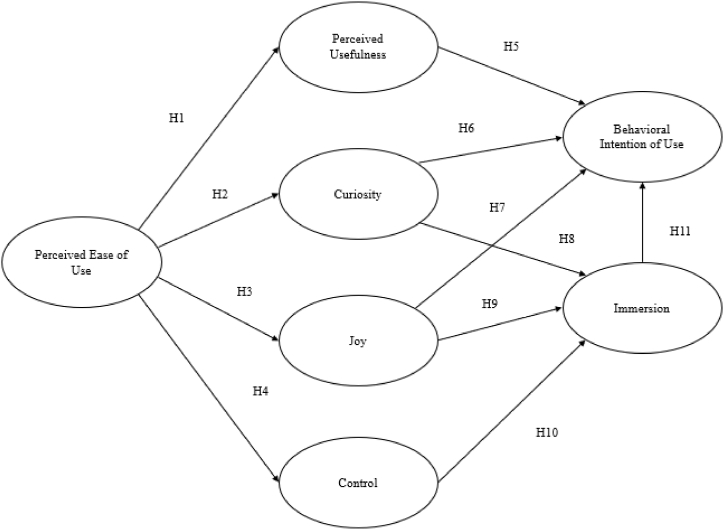
Proposed research model of Study 1.

The following Fig. 2 depicts the proposed research model of Study 1.

#### 3.2.2 Measurement

Questionnaire items were adapted from the Hedonic Motivation Use Acceptance Scale developed by Ref. [43]; which contains seven dimensions and 27 items, with responses rated on a five-point Likert scale (1 = strongly disagree; 5 = strongly agree). To establish reliability, we used integrated reliability (rho_A), which is a more accurate measure of reliability than Cronbach's alpha, as the former does not assume that the project loads or error terms are equal (Chin et al., 2003). Each framework in the research model met the standard minimum threshold of 0.7. The validity of this questionnaire was examined using composite reliability (CR), average variance extracted (AVE), and the heterotrait-monotrait ratio (HTMT). Appendix 1 presents details regarding the reliability and validity of the questionnaire. As both independent and dependent variables were measured in one survey using the same response method, the issue of common method variance (CMV) may occur. Study 1 applied Harman's One-Factor Test to examine the potential threat of CMV [44]. The first unrotated factor captured 36 % of the variance in the data, indicating that CMV was not an issue in Study 1 [45].

### 3.3 Study 2

Study 2 was a within-subject experiment which aimed to provide empirical evidence to cross-examine the results of Study 1. In Study 2, each participant experienced two settings (VR- and non-VR-based language learning), and the dependent variable was EFL learners' CA measured using electroencephalogram (EEG), which is a valid approach for such measurement [46]. A prior study of [47] had adopted EEG to detect participants’ emotional engagement (which was considered as cognitive absorption) in AR/VR for virtual tourism. An EEG electrode was placed in the F3 position (the nearest position to the dorsolateral prefrontal cortex) and is related to cognitive activities [48]. EEG enables the detection of brain activity in response to cognitive stimuli (Anderson & Bratman, 2008; [49]. The information stemming from oscillatory frequency induces neural oscillations, which proposed that brain wave activities can be considered “the alphabet for brain functions” (Antonenko et al., 2010; Basar, 1980). Among the information stemming from the power spectrum density, alpha brainwaves (8–13 Hz) are positively related to CA, whereas a negative relationship exists between beta brainwave (13–30 Hz) and CA [46]. Therefore, Study 2 used α/β ratio as the CA index, with higher scores indicating greater sense of perceived CA. To ensure the quality of EEG data, Study 2 was administered in a laboratory, which was soundproof with consistent lighting. Their learning outcomes were assessed with a series of comprehension questions about the contents that they had just learned at VR- and non-VR-based language learning settings.

#### 3.3.1 Participants and design

Study 2 addressed RQ2 and RQ3. It was conducted in a laboratory on a one-on-one basis. The number of participants was determined using G*Power (effect size = 0.25, α error probability = .05, statistical power = .8), which suggested a sample of 27 for a matched pair *t*-test. Thus, a purposeful sampling technique was employed to recruit participants from the campus of a public university in southern Taiwan. To represent most Taiwanese students at the postsecondary level, individuals with Test of English for International Communication (TOEIC) scores of approximately 568, which was the average score of all Taiwanese TOEIC takers in 2021 (ETS, 2021), were included. Thirty-three Taiwanese EFL learners were recruited. Their demographic traits included gender (18 women, 54.54 %; 15 men, 45.45 %) and age (*M* = 20.6, *SD* = 1.2) and all of them were from the same academic department, which was hospitality management.

The study was conducted in a laboratory where environmental factors, such as lighting and noise, were controlled. The experiment was conducted individually. All participants provided written informed consent before participating, and they experienced two settings (VR- and non-VR-based language learning), each session lasting 10 min (please see Fig. 4 for the detailed experiment process). Contents of the subject matter were developed and designed by the research team, which focused on tourism-related English. Contents of this VR-based language learning were shown in a real international airport (Kaohsiung International Airport, Taiwan), wherein scenes of this VR-based language learning displayed different parts of the airport, including check-in counters, security checkpoints, immigration counters, and duty-free stores. What the participants saw in the HMD included the scenes, queues and conversations that usually occurred at those specific points. For instance, at the security checkpoint, the sentence ‘Please walk through the metal detector’ was shown. It had been confirmed that the participants were not familiar with these contents as they either had never been abroad or had not been abroad for the past three years because of COVID. The levels of difficulty of the content of the two sessions were identical. In terms of the variables of this experiment, the setting was the independent variable, and the participants' CA was the dependent variable. Subsequently, the participants' learning outcomes were measured using the content they had learned. In this phase, the independent variable was learners' CA, and the dependent variable was the learning outcome. Study 2 was designed and implemented in accordance with the Declaration of Helsinki and approved by the Institutional Research Board (NCKU HREC 110–577). The procedure of this experiment is depicted in Fig. 4, and the contents of VR- and non-VR-based language learning are presented in Fig. 5, Fig. 6, respectively.

#### 3.3.2 Measurement

The participants’ CA was measured using alpha and beta brainwaves at the prefrontal cortex. EEG was collected and recorded using an EEG sensor with an Infinity encoder manufactured by Thought Technology, as described by Ref. [46]. Electrodes were placed on the prefrontal cortex (F3 and F4 position of 10–20 system, please see Ref. [50] for details). EEG data were obtained while the participants were using VR-based language learning. EEG data retrieved during resting periods were subtracted, as they serve as baseline data, as suggested by Ref. [25]. With respect to their learning outcomes, they were asked a comprehension question about the contents that they just learned with/without VR-based language learning. Their performance was judged on a scale of 10 by two examiners who had been EFL instructors at higher educational institutes in Taiwan for more than 10 years. Inter-rater reliability kappa was .83, which was considered as almost perfect agreement between the two adjudicators [51]. The collected data were analyzed using the *t*-test and Bayes Factor, as suggested by Ref. [52] to examine two research hypotheses of Study 2.

RH12 Would EFL learners' cognitive absorption be significantly different in VR and non-VR-based language learning settings?

RH13 Would EFL learners' various levels of cognitive absorption lead to significantly different learning outcomes?

In terms of the statistical techniques being adopted to examine the research hypotheses of Study 2, the classical frequentist statistics (null hypothesis testing mindset) as well as Bayesian statistics (model checking mindset) would be used for this study to acquire both the ‘deductive process of computing probabilities given certain parameters of probability distributions (like the mean and variance of a normal distribution)’ as well as ‘the inductive process of “guessing” best choices for parameters’ [53]; p. 1).

#### 3.3.3 Manipulation check

The participants were asked to rate how immersed they were in the two settings they experienced on a five-point scale. The results show that the participants generally perceived different levels of immersion in these two settings (*M*_*VR*_ = 4.12, *SD* = 0.56, *M*_*no VR*_ = 3.12, *SD* = 0.51, *t*(32) = 7.584, *p* < .001, Cohen's *d* = 1.867. Therefore, it was reasonable to conclude that the manipulation was successful.

## 4 Results

### 4.1 Results of study 1

**Fig. 3:**
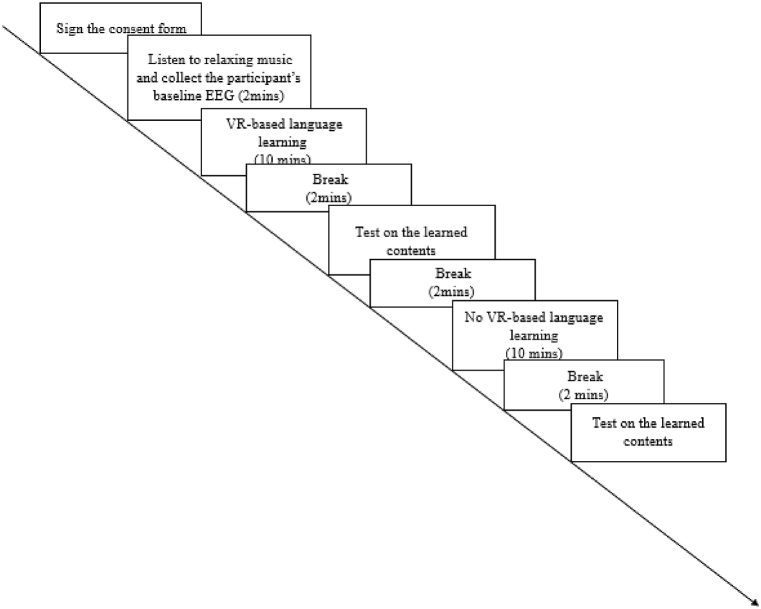
Experiment procedure of Study 2.

PLS-SEM analysis reports three essential pieces of information: path coefficient (*β*), R-squared (*R*^*2*^), and corresponding *t*-values (Hair et al., 2017). The PLS-SEM results indicated that 8 out of 10 proposed research hypotheses were significant at the 0.05 level, except for the construct of curiosity (Fig. 3). Perceived ease of use was significantly associated with perceived usefulness (*β* = 0.735, *t* = 19.619, CI 95 % [0.658, 0.804]), curiosity (*β* = 0.695, *t* = 20.544, CI 95 % [0.629, 0.763]), joy (*β* = 0.706, *t* = 19.805, CI 95 % [0.634, 0.774]), and control (*β* = 0.723, *t* = 20.346, CI 95 % [0.650, 0.790]). Perceived usefulness and joy significantly influenced EFL learners' behavioral intentions to use VR to learn English (*β* = 0.345, *t* = 3.811, CI 95 % [0.153, 0.510] and *β* = 0.340, *t* = 4.101, CI 95 % [0.179, 0.503], respectively), with perceived usefulness having a stronger effect than joy. Furthermore, EFL learners' joy and sense of controlling VR significantly influenced immersion in VR settings (*β* = 0.262, *t* = 2.761, CI 95 % [0.076, 0.453] and *β* = 0.522, *t* = 6.648, CI 95 % [0.360, 0.671] respectively), with control having a stronger effect than joy. Curiosity had a significant association with behavioral intention (*β* = 0.131, *t* = 2.075, CI 95 % [0.009, 0.254]; however, its effect on immersion was not significant. The sense of immersion created by VR also had a positive and significant effect on EFL learners’ behavioral intention of using VR-based language learning (*β* = 0.185, *t* = 2.609, CI 95 % [0.043, 0.324]). The lowest adjusted *R*^*2*^ value among all constructs was 0.481, indicating that the independent variable explained at least 48.1 % of the variance in the dependent variable. Table 2 reports the results of research hypotheses of Study 1 stemmed from PLS-SEM.

**Fig. 4:**
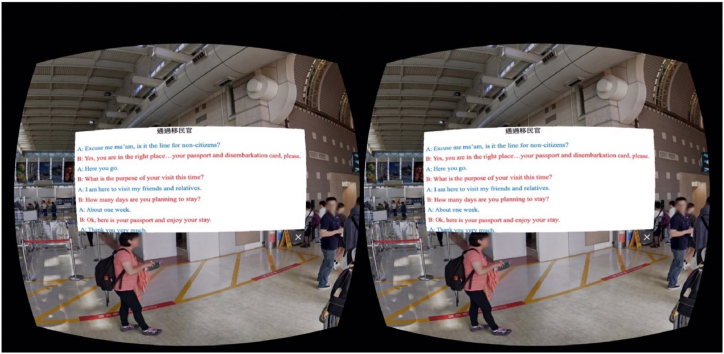
VR-based language learning setting.

**Fig. 5:**
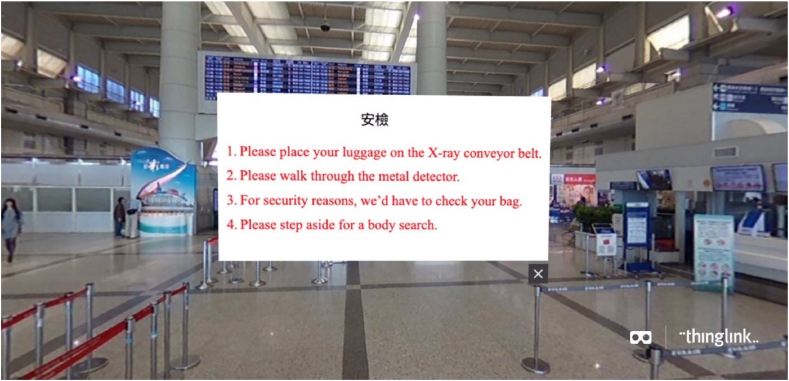
No VR language learning setting.

**Fig. 6:**
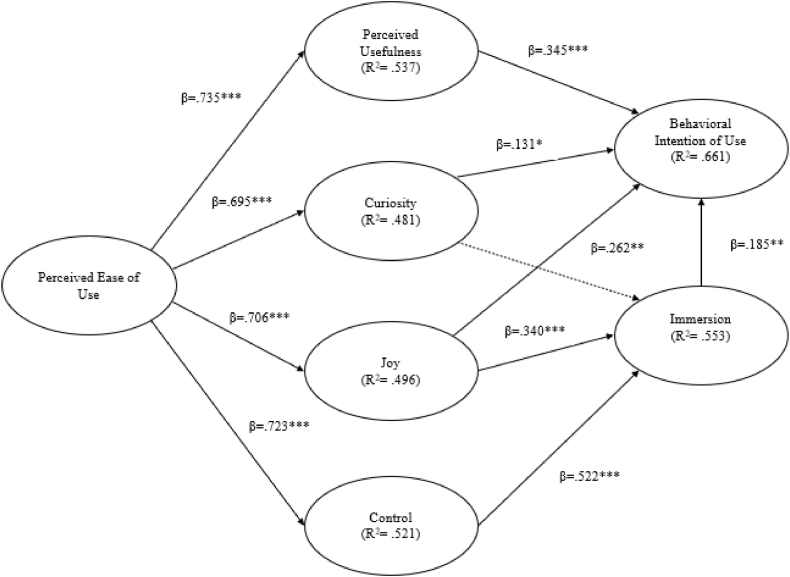
Results of PLS-SEM

**Table 2:** Results of hypotheses examination of Study 1.

Research Hypothesis	Result
Perceived Ease of Use → Perceived Usefulness	Supported
Perceived Ease of Use → Curiosity	Supported
Perceived Ease of Use → Control	Supported
Perceived Ease of Use → Joy	Supported
Perceived Usefulness → Behavioral Intention to Use	Supported
Curiosity → Behavioral Intention to Use	Supported
Joy → Behavioral Intention to Use	Supported
Curiosity → Immersion	Not Supported
Joy → Immersion	Supported
Control → Immersion	Supported
Immersion → Behavioral Intention to Use	Supported

### 4.2 Results of study 2

Study 2 revealed a significant difference in the CA index between the two sessions. Specifically, the each participant's CA was significantly higher in the VR-than non-VR-based language learning setting (*t*(32) = 9.750, *p* < .001, Cohen's *d* = 2.592). This result was cross-examined using the Bayes Factor (*BF* = 0.000), which indicated no support for the null hypothesis [54]; therefore, the frequentist and Bayesian statistics agreed with this result. Furthermore, the results indicated that various levels of CA led to significantly different learning outcomes, and participants achieved better learning outcomes when they were placed in a VR-than non-VR-based language learning setting (*t*(32) = 4.476, *p* < .001, Cohen's *d* = 1.005), and these results were supported by Bayesian statistics (*BF* = 0.004). Table 3 reports the results of Study 2.

**Table 3:** Results of study 2.

	*Mean*	*SD*	*t*	*p*	Cohen's *d*
Participants' CA in VR-based language learning setting.	1.161	.298	9.750	.000	2.592
Participants' CA in non-VR-based language learning setting.	.998	.157			
Participants' learning outcomes in VR-based language learning setting.	4.333	1.164	4.476	.000	1.005
Participants' learning outcomes in non-VR-based language learning setting.	3.242	1.001			

## 5 Discussion

### 5.1 Study 1

The results of Study 1 answered RQ1 (What are the variables that affect EFL learners' acceptance of VR-based language learning?) and echoed earlier studies, such as [43]; providing further insightful information. Generally, the proposed 11 research hypotheses were supported by the PLS-SEM results; accordingly, applying HMSAM to also investigate the factors that can predict EFL learners' use of VR-based language learning is a sound approach. One interesting yet insightful finding of Study 1 mostly come from the significant role of curiosity in behavioral intention to use; nevertheless, its effect on the sense of immersion in VR-based language learning settings was not significant. The former aligns with the findings of [41,55] but differs from those of Trapero's study (2018), which argued that an individual's curiosity about AR is not a significant predictor of their behavioral intention to use such technologies in educational contexts. A possible explanation for this discrepancy may be the orientation toward the use of emerging technologies, as [41,55] used VR as a tool for digital game-based learning, whereas [56] investigated a mobile augmented reality learning application. Regarding the insignificant effect that curiosity had on immersion entailed by VR could be understood in the context that most participants were digital natives [57] who grew up in a technology-savvy age. The short video depicting VR-based language learning shown to the participants before they did the survey might not be that immersive in comparison to the VR-based commercial games they were familiar with. In the future, when students who are “virtual natives” (Henry & Shannon, 2023) come to classes, the educational landscape will also be different. Subsequently, immersion of VR did significantly affect EFL learners' behavioral intention of using VR-based language learning, as shown by Study 1. Even though ‘it is very important to note that “high” does not necessarily equate to “good,” and “low” is not meant to imply “bad”’ [4]; p. iv), results of Study 1 posited that immersion was indeed a significant predictor of EFL learners' intention to use VR for English learning.

To come to the point, it is to the best knowledge that this study was the first to adopt the HMSAM to explore EFL learners' perceptions of VR-based language learning. Furthermore, this online survey only revealed EFL learners' hedonic and motivation system adoption of VR-based language learning, whereas underlying reasons remained unknown. As such, Study 2 was designed and conducted to take one step further to investigate the physiological mechanism of EFL learners’ using VR-based language learning from the perspective of cognitive absorption.

### 5.2 Study 2

The results of Study 2 provided empirical evidence that VR technology enhanced learners' CA effectively, which was in line with previous studies [58]. Furthermore, Study 2 demonstrated the validity of this argument for EFL learners. However, absorption in VR may not be advantageous [26]. stated that people (i.e., language learners) were “extensively absorbed in the technology at the expense of language use” (p. 1993). As such, participants’ learning outcomes were examined in Study 2. The results indicated that CA led to different learning outcomes for EFL learners, which echoed previous research that reported better learning outcomes resulting from VR technology [59,60]. Specifically, participants showcased better learning outcomes with VR-based language learning, and this finding is in line with recent research of [61] and that of [62]. As such, it is sound for Study 2 to provide neurophysiological evidence to support the effectiveness of VR-based language learning.

### 5.3 Implications and limitations

With the increasing advancement of technology, an increasing number of educators regard immersion language learning as a complex social activity with strong contextualized, embodied, and experiential features. Thus, teachers must pay attention to and consider language learning and teaching from the perspective of cultivating students’ critical ability to communicate with others in real time. Immersive VR technology can help teachers fulfill this expectation [63]. As VR technology has been widely adopted in educational practice, extensive research has been conducted to examine the effectiveness of such applications. Previous research on the application of virtual reality in foreign language learning has focused on learning effects and subjective feelings of learners; however, what causes this phenomenon or feeling remains unknown.

This study extended current understanding of the applicability of VR-based language learning through the lens of EFL learners' CA. A survey of EFL learners' hedonic motivation system adoption of VR in EFL learning was conducted using the HMSAM [43]. The disadvantages of self-reported surveys have been pointed out [64], and other approaches are expected to complement them. Neurophysiological methods could help clarify the mechanism of effective teaching, not only to determine whether a specific method is effective for education but also to understand its principles, promote the optimization of teaching methods, and improve teaching effectiveness [65]. Study 2 conducted a neurophysiological experiment to provide more insights into the effectiveness of VR-based language learning from the EFL learners' perspective of CA. This experience revealed that EFL learners’ curiosity toward VR may not be a significant predictor of their behavioral intention to use and perception of immersion, where the other constructs were significantly associated with each other. Study 2 followed [40]; who replaced the four constructs of the HMSAM (perceived usefulness, curiosity, joy, and control) with CA and found that VR increased CA among EFL learners, leading to better learning outcomes.

The findings of this study have theoretical and practical implications. The results indicated that the HMSAM could also be used to explain VR-based language learning contexts. However, EFL learners' curiosity was not a valid predictor of the sense of immersion. Today, learners are used to emerging technologies, and VR technologies may not inspire curiosity, as many digital games are played on VR-based platforms. Nevertheless, the development of innovative technology has been beyond expectations, and emerging technologies may have negative consequences, such as anxiety about new technologies [66]. Future studies should explore this issue in greater detail. Furthermore, this study confirmed that VR boosted EFL learners’ CA. This finding provides empirical support for previous claims regarding the relationship between VR technology and technology-enhanced language learning based on neurophysiological experiments, indicating that different levels of CA lead to significantly varying learning outcomes. However, the underlying reasons for the influence of CA on learning outcomes remain unclear. Therefore, future studies should address this issue. This study supports the practical implications of the concept of VR-based language learning.

Using a survey and experiment, this study provided a holistic picture of the applicability of VR technology in language learning. However, this study has some limitations. Participants who took part in the survey were required to have prior experience using VR to learn other subjects; however, learning a foreign language is different from learning other subjects, such as physics and geography [67]. Whether it is suitable to transfer their learning experience with VR to VR-based language learning remains to be clarified. Another weakness of this present research would be the level of diversity among the research participants. In the experiment, although the number of participants met the minimum required number calculated with G*Power, more culturally diverse participants should be included [68]. indicated that cultural differences were significant moderators of learners' e-learning adoption. A broader spectrum of participants' neuronal mechanisms drawn from culturally diversified samples may help them acquire a better understanding when using VR-based language learning. Last but not least, learning outcomes were measured immediately after the intervention of the experiment; hence, these learning outcomes related to working memory capacity, which could be moderated by other factors, such as learning contexts and learners’ proficiency in English [69], and may be temporary. Future research should include a delayed test or a longitudinal investigation to understand the long-term effects of VR technologies on EFL learning.

## 6 Conclusion

Two studies were conducted to explore the CA of EFL learners in VR-based language learning. CA is the state of deep involvement and enjoyment in an activity. The first study was a survey that used the HMSAM to measure the factors that influence EFL learners' intention to use VR. The results showed that CA was the principal factor, followed by perceived enjoyment and usefulness. The second study was a neurophysiological experiment that used EEG to measure the brain activity of learners while they were engaged in VR-based language learning. The results demonstrated that the VR group had higher CA than the control group. These findings suggest that VR enhanced EFL learners' CA and motivation for language learning. Moreover, EEG data revealed that the VR group had greater activation in the frontal and temporal regions of the brain, which are associated with language processing and social cognition. These findings suggest that VR enhanced EFL learners' CA by providing an immersive and realistic experience that stimulated language and social skills. Immersive VR technology could help teachers fulfill the expectations of cultivating students' critical ability to communicate with others in real time, as it creates a complex social activity with strongly contextualized, embodied, and experiential features. Thus, future research should explore the long-term effects of VR-based language learning on learners’ language proficiency and motivation.

## Data availability statement

There has no data associated with this present study been deposited into a publicly available repository and data will be available on request.

## CRediT authorship contribution statement

**Liwei Hsu:** Writing - review & editing, Writing - original draft, Visualization, Validation, Supervision, Software, Resources, Project administration, Methodology, Investigation, Funding acquisition, Formal analysis, Data curation, Conceptualization.

## Declaration of competing interest

The authors declare the following financial interests/personal relationships which may be considered as potential competing interests:Liwei Hsu reports administrative support and equipment, drugs, or supplies were provided by National Kaohsiung University of Hospitality and Tourism. Liwei Hsu reports a relationship with National Science and Technology Council, Taiwan that includes: funding grants. If there are other authors, they declare that they have no known competing financial interests or personal relationships that could have appeared to influence the work reported in this paper.
