# Supplementary material for: Exploring EFL learners’ acceptance and cognitive absorption at VR-Based language learning: A survey and experimental study
Source: Heliyon. 2024 Jan 20;10(3):e24863. doi: 10.1016/j.heliyon.2024.e24863 (PMC10838755; doi:10.1016/j.heliyon.2024.e24863)
Supplement: Multimedia component 1 [file mmc1.docx]

| Construct | Indicator | Item | Factor Loading | Cronbach’s Alpha | CR | rho_A | AVE |
| --- | --- | --- | --- | --- | --- | --- | --- |
| Perceived ease of use (PE) | PE1 | I think it is easy to use VR to learn English. | .89 | .94 | .95 | .94 | .73 |
|  | PE2 | I think the interaction with VR is clear and understandable. | .90 |  |  |  |  |
|  | PE3 | I think it easy to get VR to do what I want them to do. | .85 |  |  |  |  |
|  | PE4 | I believe Interacting with VR does not require a lot of mental effort. | .87 |  |  |  |  |
| Perceived usefulness (PU) | PU1 | I think using VR would improve my English learning. | .89 | .89 | .89 | .92 | .69 |
|  | PU2 | I think using VR would enhance my learning effectiveness. | .88 |  |  |  |  |
|  | PU3 | I think using VR to learn would enhance my productivity. | .84 |  |  |  |  |
|  | PU4 | I believe VR would be a useful tool in my English learning. | .83 |  |  |  |  |
| Behavioral intention towards use of VR (BIU) | BIU1 | O would consider VR makes English learning more interesting. | .84 | .89 | .89 | .92 | .69 |
|  | BIU2 | I look forward to English lessons that require me to use VR. | .81 |  |  |  |  |
|  | BIU3 | I think learning English with VR is fun. | .88 |  |  |  |  |
|  | BIU4 | I like the idea of using VR for English learning | .83 |  |  |  |  |
| Joy of using VR for learning English (JOY) | Joy1 | I think the learning experience with VR-based English lessons to be enjoyable | .85 | .88 | .90 | .91 | .64 |
|  | Joy2 | I think I will fun using VR for the English learning. | .86 |  |  |  |  |
|  | Joy3 | I do not agree that using the VR was boring. | .90 |  |  |  |  |
|  | Joy4 | I think VR-based English learning would make me satisfied. | .81 |  |  |  |  |
| Control over VR (CTL) | CTL1 | I think I would have a lot of control of VR. | .88 | .90 | .91 | .92 | .72 |
|  | CTL2 | I think I would be in control of VR. | .90 |  |  |  |  |
|  | CTL3 | I do not think I would have no control over my interaction with VR. | .90 |  |  |  |  |
|  | CTL4 | I think I would be allowed to control my interaction on VR. | .81 |  |  |  |  |
| Focused Immersion on VR-based English learning (FI) | FI1 | I think I would be able to block out most other distractions. | .85 | .90 | .91 | .93 | .77 |
|  | FI2 | I believe I would be absorbed in what I will be doing. | .91 |  |  |  |  |
|  | FI3 | I think I would be immersed in the use of VR. | .92 |  |  |  |  |
|  | FI4 | I do not think I would be distracted by other attentions very easily. | .85 |  |  |  |  |
| Curiosity on VR-based English learning (CUR) | CUR1 | This experience would excite my curiosity. | .91 | .89 | .89 | .93 | .82 |
|  | CUR2 | This experience would make me curious | .92 |  |  |  |  |
|  | CUR3 | This experience would arouse my imagination | .89 |  |  |  |  |

Discriminant Validity of Questionnaire with Heterotrait-Monotrait Ratio (HTMT)

|  | BIU | CLT | CUR | FI | JOY | PEOU | PU |
| --- | --- | --- | --- | --- | --- | --- | --- |
| BIU |  |  |  |  |  |  |  |
| CLT | .79 |  |  |  |  |  |  |
| CUR | .73 | .71 |  |  |  |  |  |
| FI | .73 | .79 | .56 |  |  |  |  |
| JOY | .82 | .78 | .82 | .72 |  |  |  |
| PEOU | .71 | .78 | .76 | .62 | .76 |  |  |
| PU | .86 | .82 | .75 | .76 | .86 | .80 |  |

*Note: BIU=Behavioral Intention of Use, CLT=Control, CUR=Curiosity, FI=Focused Immersion, J=Joy, PEOU=Perceived Ease of Use, PU=Perceived Usefulness*
